# Stable sulforaphane protects against gait anomalies and modifies bone microarchitecture in the spontaneous STR/Ort model of osteoarthritis

**DOI:** 10.1016/j.bone.2017.07.028

**Published:** 2017-10

**Authors:** Behzad Javaheri, Blandine Poulet, Ahmed Aljazzar, Roberto de Souza, Miriam Piles, Mark Hopkinson, Elaine Shervill, Andrea Pollard, Boris Chan, Yu-Mei Chang, Isabel R. Orriss, Peter D. Lee, Andrew A. Pitsillides

**Affiliations:** a Skeletal Biology Group, Comparative Biomedical Sciences, The Royal Veterinary College, Royal College Street, London, NW1 0TU, UK; b Institute of Ageing and Chronic Disease, University of Liverpool, West Derby Street, Liverpool L7 8TX, UK; c Universidade Federal de Mato Grosso (UFMT), Departamento de Clínica, Cuiabá, Brazil; d Department of Orthopaedics & Traumatology, The University of Hong Kong, Pok Fu Lam, Hong Kong; e Manchester X-Ray Imaging Facility, University of Manchester, Manchester, UK

**Keywords:** Osteoarthritis, Sulforaphane, SFX-01, Cartilage, STR/Ort

## Abstract

Osteoarthritis (OA), affecting joints and bone, causes physical gait disability with huge socio-economic burden; treatment remains palliative. Roles for antioxidants in protecting against such chronic disorders have been examined previously. Sulforaphane is a naturally occurring antioxidant. Herein, we explore whether SFX-01®, a stable synthetic form of sulforaphane, modifies gait, bone architecture and slows/reverses articular cartilage destruction in a spontaneous OA model in STR/Ort mice. Sixteen mice (*n* = 8/group) were orally treated for 3 months with either 100 mg/kg SFX-01® or vehicle. Gait was recorded, tibiae were microCT scanned and analysed. OA lesion severity was graded histologically. The effect of SFX-01® on bone turnover markers *in vivo* was complemented by *in vitro* bone formation and resorption assays. Analysis revealed development of OA-related gait asymmetry in vehicle-treated STR/Ort mice, which did not emerge in SFX-01®-treated mice. We found significant improvements in trabecular and cortical bone. Despite these marked improvements, we found that histologically-graded OA severity in articular cartilage was unmodified in treated mice. These changes are also reflected in anabolic and anti-catabolic actions of SFX-01® treatment as reflected by alteration in serum markers as well as changes in primary osteoblast and osteoclast-like cells *in vitro*. We report that SFX-01® improves bone microarchitecture *in vivo,* produces corresponding changes in bone cell behaviour *in vitro* and leads to greater symmetry in gait, without marked effects on cartilage lesion severity in STR/Ort osteoarthritic mice. Our findings support both osteotrophic roles and novel beneficial gait effects for SFX-01® in this model of spontaneous OA.

## Highlights

• SFX-01® treatment improves bone microarchitecture *in vivo* in STR/Ort mice.
• SFX-01® treatment produces corresponding changes in bone cell behaviour *in vitro*.
• SFX-01® leads to greater symmetry in gait, without marked effects on cartilage lesion.

## 1 Introduction

Osteoarthritis (OA) has major joint and bone hallmarks, and is the most common cause of gait impairment and physical disability [1]. It is clear that OA has major genetic and mechanical determinants, yet its complex aetiology means that its features remain poorly understood. OA affects all facets of joint integrity, with thinning of the articular cartilage accompanied by subchondral bone thickening, along with formation of bony osteophytic projections [2]. The role of bone, especially the subchondral plate, in the initiation and progression of OA has been the subject of many studies [3], [4], [5], [6]. Clinical studies have reported an elevation in indices of bone formation and resorption [7], suggesting that bone remodelling is enhanced in OA patients. Despite such improvement in our understanding of OA, effective treatment options remain limited. The significant socio-economic burden this creates, requires development of new disease-modifying drugs which effectively target OA changes in cartilage and bone.

To identify OA-modifying drug targets, various mouse OA models have been used [8], [9], including: models in which OA arises spontaneously, such as in STR/Ort mice [9], [10]. Male STR/Ort mice develop a natural OA, aged ∼ 20 weeks that engages processes resembling those seen in human OA, with a loss of articular cartilage proteoglycan, progressive articular cartilage degeneration, osteophytogenesis, and subchondral bone thickening [10], [11], [12]. Methods for monitoring OA severity in STR/Ort mice include cartilage lesion grading after termination [13] as well as non-invasive monitoring of gait [14], which has been linked with OA severity and knee pain in humans [15], [16]. It has also been reported that STR/Ort mice have high bone mass, however, how this is related to OA progression remains to be clarified [17], [18].

Use of antioxidants in the prevention or reversal of chronic disorders, including OA, has been studied extensively. One indirect antioxidant, sulforaphane (SFN), is a naturally-occurring potent modulator of phase II xenobiotic-metabolizing enzymes. SFN is found at high concentrations in precursor form, as glucoraphanin, in cruciferous vegetables, particularly broccoli (*Brassica oleracea* italica) [19] and is released by hydrolytic myrosinase activity. SFN (1-isothiocyanato-4-methylsulphinylbutane) activates the transcription factor NF-E2–related factor 2 (Nrf2), which binds to an antioxidant response element in cognate genes. SFN is rapidly absorbed by humans and previous studies have reported anti-inflammatory effects [20]. It has recently emerged that SFN also protects retinal pigment epithelial cells, pancreatic islet β-cells, kidney, skeletal muscle, vascular smooth muscle cells, cardiomyocytes and neurons against oxidative stress.

SFN also modulates expression of matrix metalloproteinases in chondrocytes [21] and has been reported to regulate osteoclastogenesis by inhibiting receptor activator of nuclear factor-κB ligand (RANKL) [22]. An exciting recent study extended these effects of SFN to the protection of articular cartilage destruction [23]. These authors found that administration of a SFN-rich diet significantly reduced cartilage destruction 12 weeks after induction of OA by surgical destabilisation of the medial meniscus (DMM) [23]. It was acknowledged, however, that surgically induced OA is not necessarily an ideal model in which to assess the protective role of SFN, and that SFN is inherently unstable, rendering it a nonviable pharmaceutical agent.

Indeed, SFN is known to degrade in response to heat, is sensitive to light, oxygen and is affected by pH changes [24], [25]. Recently, SFN was stabilised by formation of a synthetic sulforaphane-alpha-cyclodextrin inclusion complex, leading to the generation of a stable compound, named Sulforadex (SFX-01®), which has provided the opportunity to utilize the full potential of SFN. In the present study, we have explored whether SFX-01® slows and/or reverses the destruction of cartilage in the STR/Ort model of spontaneous OA. To achieve this, we use both non-invasive monitoring of gait modifications, as well as extensive post-mortem analysis of joint and bone disease hallmarks to examine how OA in these mice is influenced by *in vivo* SFX-01® treatment. Finally, the effect of SFX-01® on mouse osteoblast and osteoclast function was assessed using established *in vitro* protocols.

## 2 Materials and methods

### 2.1 Animals

Male STR/Ort mice were housed in a facility at 21 ± 2 °C with 12-hour light/dark cycles at the Royal Veterinary College (RVC) in polycarbonate or polypropylene cages with wood chip and paper bedding. The mice were housed up to 4 per cage; weaners up to 8 weeks of age were fed a standard rodent breeding diet and thereafter a standard rodent maintenance diet (Special Diet Services, South Witham, UK). All of the procedures conducted in the facility were in accordance with the Animals Act (Scientific Procedures) 1986 and local RVC ethical guidelines.

### 2.2 SFX-01® treatment

At 26 weeks of age, sixteen male STR/Ort mice (*n* = 8/group) were assigned randomly to vehicle and SFX-01® groups and were treated orally for 3 months with daily administration of either 100 mg/kg SFX-01® (a stable form of SFN, PharmAgra Labs, CN, USA) or vehicle, 0.5% sodium carboxymethyl cellulose (Sigma, Poole, Dorset, UK) in H_2_O.

### 2.3 Gait analysis

Gait was recorded using a treadmill-based DigiGait™ Imaging system (Mouse Specifics Inc., Boston, MA) [26] before sacrifice. Briefly, vehicle and SFX-01® treated mice (8 per group) ran on a transparent flat treadmill at 17 cm/s, while a video camera captured ventral images. Animals ran for a maximum of 30 s, with segments of 5 s (which corresponded to > 10 consecutive strides) used for analysis. No habituation procedures were used for these studies at any time. DigiGait image analysis software automatically defines each paw area, generates waveforms to describe advance/retreat of each limb in consecutive strides, and identifies periods of time when each paw is in treadmill contact as stance phase, and intervening periods as swing phase. Postural and kinematic gait measurements were also calculated according to manufacturers' instructions. Symmetry indices, symmetry ratios, compensation and balance between the contralateral fore and the hind limbs were computed [27]. The minimum and maximum of these asymmetry measures were recorded as primary and secondary descriptors.

From those gait descriptors symmetry and balance indexes were calculated as:

$$\text{Symmetry index}\;(\mathrm{SI};100*\left(|R-L|\right)/\left[0.5*\left(R+L\right)\right]$$

Symmetry ratios:

¾ Between Hind and Fore Limbs in the same (right and left) side

¾ Between both fore limbs and between both hind limbs

Compensation or balance between the contralateral fore and the hind limbs:

¾ Between the right-hind and left fore-limbs: dgr = | RR-LF |

¾ Between the left-hind and right fore-limbs: dgl = | LR-RF |

where RR, LR, RF, LF referred to right-rear, left-rear, right-fore and left-fore limbs. The minimum and the maximum of those values, as well as the minimum of their ratio were computed. In total, there were 262 different gait descriptors. These measures of asymmetry allow for the unpredictable targeting of OA to specific hind-limb knee joints in this strain of STR/Ort mouse to be monitored. Greater symmetry indicates more ‘normal’ gait pattern (with the proviso that alternatively both limbs may be equally affected).

Mouse treadmill task noncompliance (*i.e.* refusal to undertake or complete the treadmill running task, which would be observed as inability or unwillingness of a mouse to take > 2 consecutive strides) was also recorded [14].

### 2.4 High-resolution micro-computed tomography (micro-CT)

#### 2.4.1 Scanning

Micro-CT scanning and analysis were performed as described previously [28]. Briefly, tibiae from vehicle and SFX-01® treated groups (*n* = 8/group) were fixed in neutral buffered formalin (NBF) and subsequently stored in 70% EtOH until scanning using the Skyscan 1172 (Skyscan, Kontich, Belgium), with x-ray tube operated at 50 kV and 200 μA, 1600 ms exposure time with a 0.5 mm aluminium filter and voxel size of 10 μm (~ 2 h scan time per sample). The slices were reconstructed using NRecon 1.6.9.4 (Skyscan, Kontich, Belgium). 2D/3D analyses were performed using CTAn 1.15.4.0 + version software (Skyscan, Kontich, Belgium). Finally, CTvox 3.1.0 r1167 version (Skyscan, Kontich, Belgium) was used for 3D visualisation and production of colour-coded images of trabecular thickness.

#### 2.4.2 Morphometrical analysis

##### 2.4.2.1 Metaphyseal/epiphyseal trabecular and subchondral cortical analysis

Appearance of the trabecular ‘bridge’ connecting the two primary spongiosa bone ‘islands’ was set as reference point for analysis of the metaphyseal trabecular bone adjacent to the epiphyseal growth plate. 5% of the total bone length from this point (toward the diaphysis) was utilised for trabecular analysis of the proximal tibia [28], [29]. Epiphyseal trabecular bone was analysed as described previously [12].

##### 2.4.2.2 Whole bone cortical analysis

Whole bone analysis was performed on datasets derived from whole CT scans using BoneJ [30](version 1.13.14) a plugin for ImageJ. Following segmentation and removal of fibula from the dataset, a minimum bone threshold was selected for each bone to separate higher density bone from soft tissues and air. This threshold was used in “Slice Geometry” within BoneJ plugin to calculate cross sectional area (CSA), second moment of area around minor axis (I_min_), second moment of area around major axis (I_max_) and mean thickness determined by local thickness in 2D (Mean Thick).

### 2.5 Histology and grading of articular cartilage (AC) lesions

Dissected knees were fixed in neutral buffered formalin, decalcified (Immunocal, Quartett, Berlin), wax-embedded and 6 μm coronal sections cut. Multiple slides (∼ 10), each containing five, 6 μm sections sampled at 120 μm intervals spanning entire joint of vehicle and SFX-01® treated STR/Ort joints (*n* = 8) were stained with Toluidine blue (0.1% in 0.1 M acetate buffer, pH 5.6) and AC lesion severity scored by the methods of Chambers et al. [31], consistent with an internationally-recognised system [13]. Briefly, grade 0: normal; grade 1: rough surface or superficial zone lesions; grade 2: lesion down to the intermediate zone; grade 3: lesions down to tidemark or loss of AC; grades 4 and 5: AC loss across between 20% and 50% or 50–80% of condylar surface; grade 6: loss with subchondral bone exposure. Grading in each joint compartment (lateral/medial, tibia/femur) allowed for a maximum (most severe) grade to be assigned in each section, and used to produce an overall ‘average’ maximum grade in each group of mice, for the entire joint and for each compartment. In addition, a mean score was produced for each joint and for each compartment and these similarly used to produce an overall ‘average’ mean grade in each group of mice.

### 2.6 Assessment of osteophyte maturity and of non-AC joint tissues

Osteophyte maturity was scored as described [32]. Briefly, multiple Toluidine blue-stained sections (as AC grading), spanning each joint were graded. Grade 0: no osteophyte; grade 1: predominantly cartilaginous; grade 2: mixed cartilage and bone; grade 3: predominantly bone with marrow spaces. Maximum scores on internal and external condylar margins were recorded.

### 2.7 Hemeoxygenase-1 (HO-1) enzyme-linked immunosorbent assay (ELISA)

Mouse ImmunoSet HO-1 ELISA (Enzo Life Sciences, Exeter, UK) was performed according to manufacturer's instructions. Briefly 96-well high-binding polystyrene microtiter plates were coated with the HO-1 capture antibody, overnight at room temperature (RT) and were blocked with blocking solution for 1 h the following day. Standards and samples were added and incubated for 1 h at RT. Following 4 washes with wash buffer, samples were incubated with detection antibody for 1 h at RT. Following another set of washes, the plate was incubated for 30 min with Streptavidin, Horseradish Peroxidase (SA-HRP) conjugate. HRP activity was revealed using 3, 3′, 5, 5′-tetramethylbenzidine (TMB) for 30 min, stop solution was applied and excitation and emission were measured at 450 nm. Amount of HO-1 protein in ng/mL was calculated using the standard curve. HO-1 protein levels were normalized to total released protein.

### 2.8 Bone biomarker analyses

Serum samples were collected at the time of sacrifice and stored at − 80 °C. Serum levels of C-terminal crosslinking telopeptide of type-I collagen (CTX-I), a marker of bone resorption, and total procollagen type 1 N-terminal propeptide (P1NP) were quantified by Serum Rat-Laps ELISA assay (Immunodiagnostic Systems Ltd., Boldon, UK), following the manufacturer's instructions.

### 2.9 Mouse osteoblast cultures

Primary cells were derived from the calvarial bones of 2–4 day old mice. Osteoblasts were obtained using methods similar to those previously described [33]. Briefly, calvariae were digested using 0.25% trypsin for 10 min, 0.2% collagenase in Hank's buffered salt solution (HBSS) for 30 min, and finally 0.2% collagenase in HBSS for 60 min, all at 37 °C. The first two digests were discarded and cells from the final digest were resuspended in α-modified essential medium supplemented with 10% foetal calf serum, 100 U/ml penicillin, 100 μg/ml streptomycin, 0.25 μg/ml amphotericin B (mixture abbreviated to ‘α-MEM’). Osteoblasts were cultured for 4 days in 75 cm^2^ flasks in a 5% CO_2_ atmosphere at 37 °C until confluent. Upon confluence, cells were plated into 6-well trays in α-MEM further supplemented with 2 mM β-glycerophosphate and 50 μg/ml ascorbate. Osteoblasts were treated with 1 nM–10 μM SFX-01® or PBS (vehicle) for the duration of the culture. Experiments were terminated by fixing the cells in 2.5% glutaraldehyde for 5 min. Cell culture plates were imaged at 800 dpi using a flat-bed scanner (Epson Perfection 4990 Photo) and the total area of bone nodules formed was quantified by image analysis, as described previously [33].

### 2.10 Mouse osteoclast cultures

The long bones were dissected from 6 week-old mice, cut across the epiphyses and the marrow was flushed out with PBS. The resulting suspension was centrifuged at 1500 rpm and resuspended in MEM supplemented with 10% foetal calf serum (FCS), 2 mM l-glutamine, 100 U/ml penicillin, 100 μg/ml streptomycin and 0.25 μg/ml amphotericin, 100 nM prostaglandin E_2_ (PGE_2_) and 50 ng/ml macrophage colony stimulating factor (M-CSF). The cell suspension was cultured for 24 h in a 75 cm^2^ flask in 5% CO_2_/95% atmospheric air to allow attachment of stromal cells and other rapidly adherent cells. The non-adherent cell suspension was removed, centrifuged and resuspended in MEM supplemented with 100 nM PGE_2_, 200 ng/ml M-CSF and 3 ng/ml RANKL (R&D Systems Europe Ltd., Abingdon, UK). Cells were plated onto 5 mm diameter ivory discs (10^6^ cells/disc) in 96-multiwells. After 24 h, discs containing adherent osteoclast precursors were transferred to 6 well trays (4 discs/well in 4 ml medium) for a further 6 days at 37 °C in 5% CO^2^/95% atmospheric air. Culture medium was acidified to pH ~ 7.0 by the addition 10 meq/l H^+^ (as HCL) on day 7 to activate osteoclasts to resorb dentine. SFX-01® (1 nM–10 μM) was added for the duration of the culture.

Osteoclasts were fixed in 2% glutaraldehyde and stained to demonstrate tartrate-resistant acid phosphatase (TRAP). Osteoclasts were defined as TRAP-positive cells with 2 or more nuclei and/or clear evidence of resorption pit formation. Osteoclast number and the area resorbed on each disc were assessed ‘blind’ by transmitted light microscopy and reflective light microscopy and dot-counting morphometry, respectively.

### 2.11 Statistical analysis

For treadmill data, Fisher's exact test was used to assess drop-out between groups. In addition, a principal component analysis was performed to extract the important information from the gait multivariate data and to express this information as a set of new uncorrelated variables (principal components, PC). For this purpose, the function PCA() from the R package “FactoMineR” was used (“R”, version 3.1.3; R Foundation for Statistical Computing, Vienna, Austria; http://www.r-project.org). A Student *t*-test (function *t*-test() from the R package “stats”) was performed to determine whether the mean of most important PC differed between both groups of animals.

A Shapiro–Wilk normality test (GraphPad Prism 6; GraphPad Software, Inc., San Diego, CA) was performed on all the datasets; all exhibited *P*-values ≤ 0.05. Two samples Student's *t*-test was applied to determine the effect of SFX-01® treatment on metaphyseal and epiphyseal trabecular and subchondral bone plate parameters and in all cases *p* < 0.05 was considered significant.

For cortical bone, graphs were developed using the R programming language “R”, version 3.1.3. Normality and homogeneity of variance of data were checked using the Shapiro-Wilk and the Bartlett's test in the R 3.1.3 respectively. Two-sample *t*-test was used to compare means between vehicle and SFX-01® treated groups. Data are expressed as mean ± SEM and values were considered statistically significant when *p* ≤ 0.05. For *in vitro* experiments, data were analysed using a one-way ANOVA, followed by a Bonferroni *post hoc* test. For all *in vitro* work, results are expressed as means ± SEM between 6 and 8 biological replicates and are representative of experiments performed at least three times using cells isolated from different animals.

## 3 Results

### 3.1 SFX-01® treatment corrects OA-associated gait asymmetry

Gait was monitored using a treadmill-based video system at 17 cm/s in vehicle and treated groups. The asymmetry index for each variable from each individual vehicle and SFX-01® treated mouse was calculated and these data used in unbiased principal component analysis aimed to examine the effect of SFX-01® treatment and to identify any other patterns. Despite multiple opportunities, 4/8 vehicle-treated and 2/8 SFX-01® treated mice failed to ever complete the treadmill task (‘drop-out’). Thus, 4 control and 6 treated mice successfully completed the treadmill task, and there was no significant difference in drop-out rate between the two groups (*p* = 0.61).

**Fig. 1:**
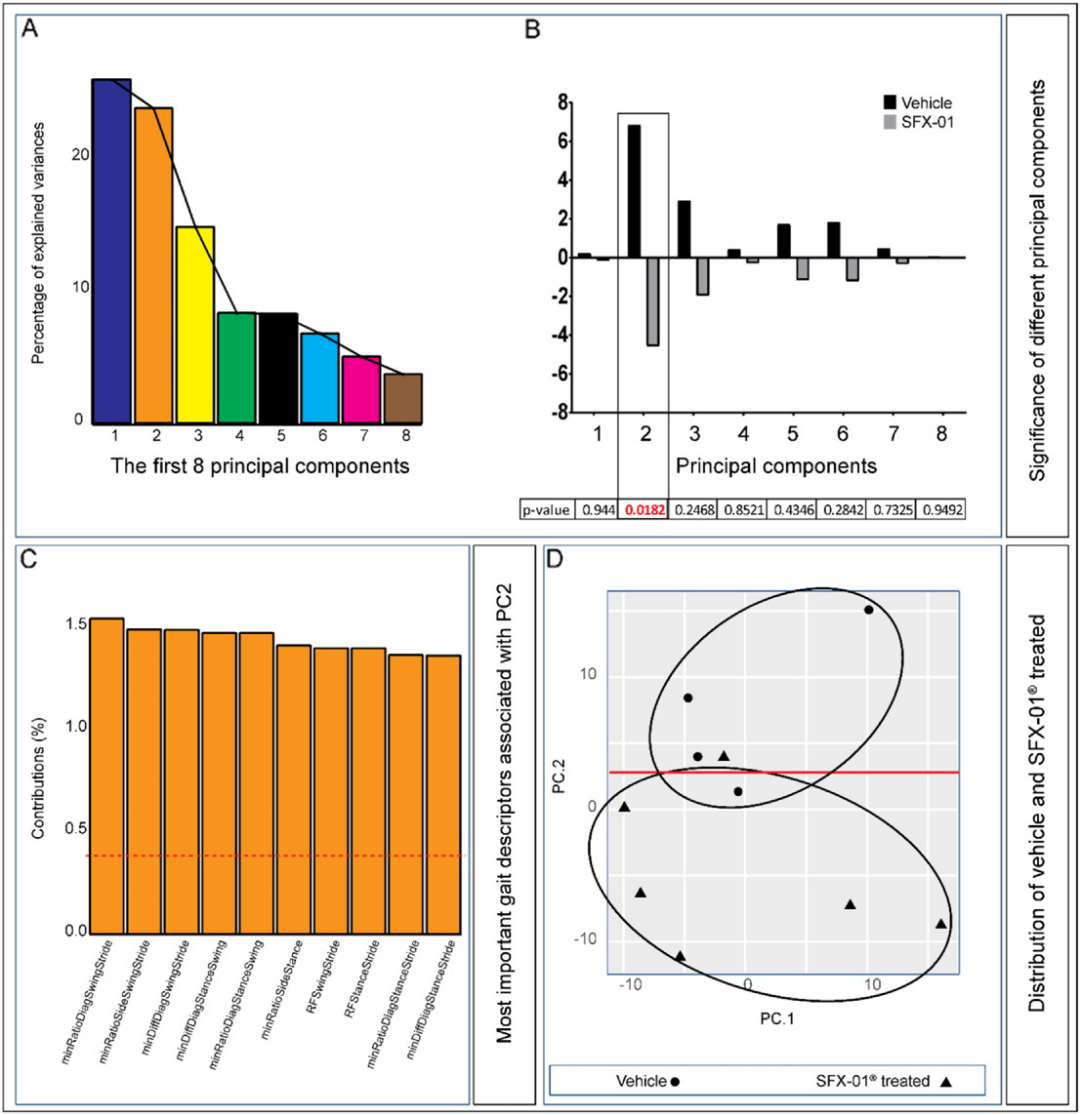
SFX-01® treatment corrects OA-associated gait asymmetry (*A*) Scree graph displaying percentage of variance explained by each of the first 8 principal components. (*B*) Average values of each of the first 8 PC's in SFX-01® (grey) and vehicle treated (black) mice and the corresponding *p*-values (beneath graph) of the differences between these groups. (*C*) The top 10 gait descriptors that associated with principal component 2 (dotted red line corresponds to the expected value if the contribution where uniform). (*D*) Scatterplot showing the significant segregation of individual vehicle (closed circles) and SFX-01® (closed triangles) treated mice on the basis of the first 2 PCs.

The importance of PC's is illustrated in Fig. 1A and indicates that 64.6% of the variation contained in the data are retained by the first three principal components and 81.1% in the first 5 PC's. Among these 8 PC's, only the second PC reached levels that were significantly different between vehicle and SFX-01® treated mice (Fig. 1B; *p* ≤ 0.05). We found that vehicle and not SFX-01® treated groups of animals clearly differ for PC2. The most important gait descriptors associated with PC2 clearly segregate the SFX-01® treated and vehicle mice and are shown in Fig. 1C. In addition, distribution of vehicle and SFX-01® treated mice are shown in Fig. 1D. Together these data indicate a significantly greater symmetry and synchronisation of movement and gait that is more rhythmic and less turbulent in SFX-01® compared with vehicle treated spontaneously osteoarthritic (control) STR/Ort mice.

### 3.2 SFX-01® treatment alters trabecular bone in both epiphyseal and metaphyseal compartments but has no effect on the tibial subchondral plate

**Fig. 2:**
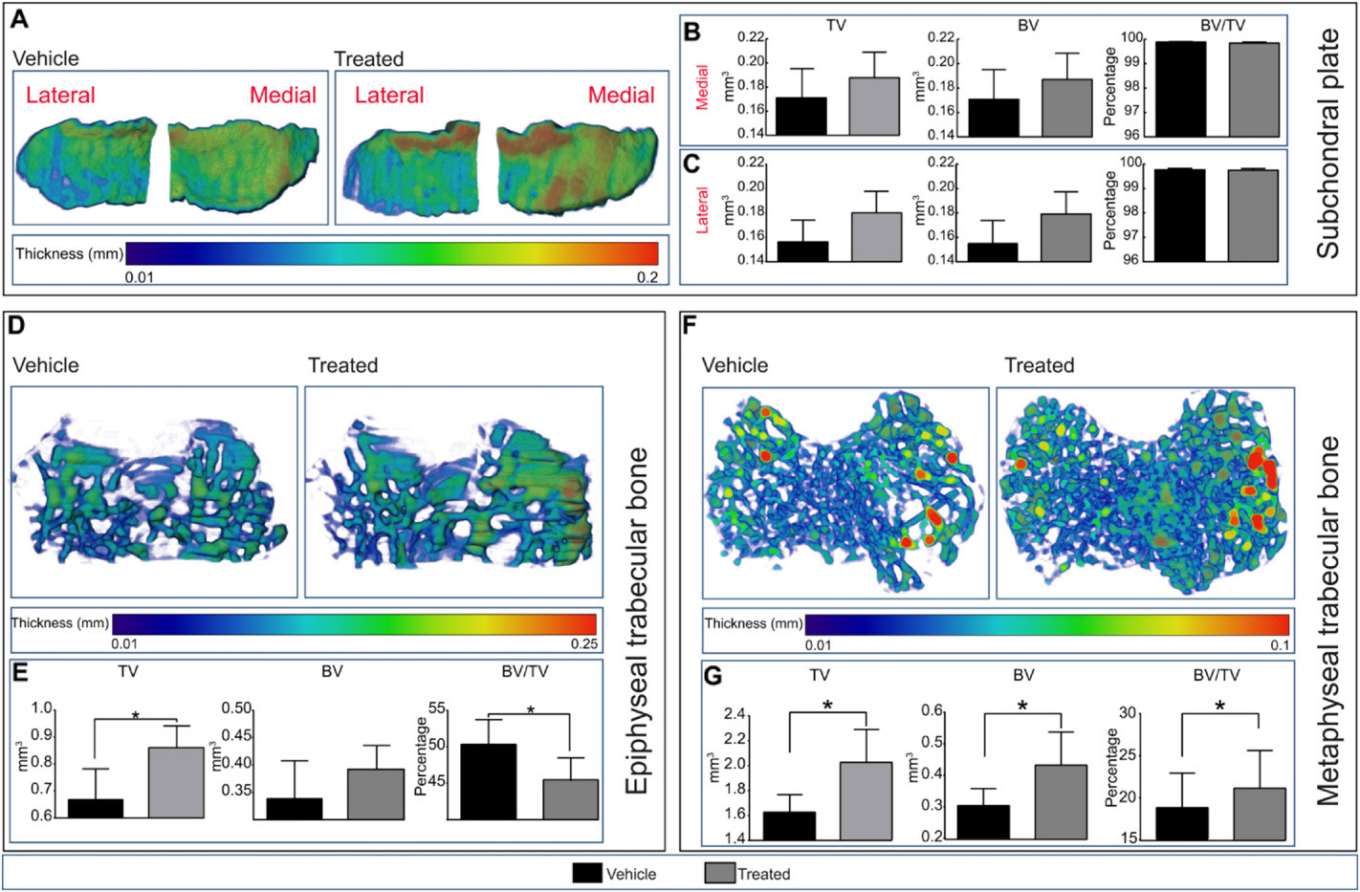
Proximal epiphyseal and metaphyseal tibial bone phenotype of vehicle (black) and SFX-01® treated (grey) mice after 11 weeks of treatment starting at 26 weeks of age. (*A*) Representative colour-coded thickness images of lateral and medial tibial subchondral plate of vehicle and SFX-01® treated mice. *(B and C)* Total volume (TV), bone volume (BV) and bone volume fraction (BV/TV) of medial (*B*) and lateral *(C)* proximal tibial subchondral plate. *(D)* Representative colour-coded thickness images of epiphyseal trabecular bone of vehicle and SFX-01® treated mouse tibia. *(E)* TV, BV and BV/TV of tibial epiphyseal trabecular bone. *(F)* Representative colour-coded thickness images of metaphyseal trabecular bone of vehicle and SFX-01® treated mice. *(g) Ex vivo* analyses to determine TV, BV and BV/TV of metaphyseal trabecular bone. Bar graphs represent means ± SEM. Group sizes were *n* = 8 for vehicle and treated mice. Statistical comparisons: * denotes *p* ≤ 0.05.

To investigate whether SFX-01® treatment modifies bone mass and architecture we examined bone in both the tibial epiphyseal and metaphyseal trabecular compartments, as well as bone of the tibial subchondral plate in vehicle and treated mice using high-resolution μCT. Analysis of both lateral and medial tibial subchondral bone plate regions revealed that subchondral plate thickness was not significantly altered in either lateral or medial tibial plateau compartments in response to SFX-01® treatment (Fig. 2A). Furthermore, we found that there was no significant effect of SFX-01® treatment on subchondral plate tissue volume (TV), bone volume (BV) and percent bone volume (BV/TV) in either compartment (Fig. 2B–C).

Epiphyseal trabecular TV was, however, significantly greater in STR/Ort mice after SFX-01® treatment (Fig. 2E; *p* ≤ 0.05) and, despite small non-significant modification in epiphyseal trabecular BV, BV/TV was significantly reduced by SFX-01® treatment in the epiphysis (Fig. 2E; *p* ≤ 0.05). However, trabecular thickness was not significantly altered in response to SFX-01® treatment (Fig. 2D). In addition to epiphyseal bone, we analysed metaphyseal bone in 5% of the total tibial length starting from the end of primary spongiosa. Our data reveal that SFX-01® treatment of STR/Ort mice also significantly increased metaphyseal trabecular TV (Fig. 2G; *p* ≤ 0.05) and, in this compartment, that this was accompanied by increases in BV (Fig. 2G; *p* ≤ 0.05) and BV/TV (Fig. 2G; *p* ≤ 0.05), with significantly higher trabecular number and mean moment of inertia (MMI) compared with the vehicle-treated group (Table 1). In agreement with the effect of SFX-01® treatment on epiphyseal trabecular bone, we found that trabecular thickness (Fig. 2F), separation and degree of anisotropy (Table 1) were also not significantly altered in this bone compartment in response to SFX-01® treatment. Together these data reveal that SFX-01® treatment of STR/Ort mice results in an expansion of the epiphyseal total volume, without any statistically significant increases in bone volume and hence a reduction in BV/TV in the epiphyseal trabecular bone compartment whilst, in contrast, treatment produces an enhancement in the trabecular total volume, bone volume and consequently bone mass in the metaphyseal compartment.

**Table 1:** Mean value of morphometric parameters representing trabecular mass and architecture of vehicle and SFX-01® treated mice. Group sizes were *n* = 8 for vehicle and treated groups. Two-sample *t*-test was used to compare means between vehicle and treated. Normality or the homogeneity of variance assumption were not violated (*p* ≥ 0.05). Data are mean ± SEM.

Morphometric index	Vehicle	Treated	*P* value
	*n* = 8	*n* = 8	Vehicle *vs* treated
Trabecular parameters			
Trabecular number (Tb.N)	2.75 ± 0.26	3.10 ± 0.47	< 0.05
Mean moment of inertia (MMI)	0.200 ± 0.02	0.275 ± 0.04	< 0.001
Trabecular separation (Tb.Sp)	0.295 ± 0.02	0.275 ± 0.05	NS
Degree of anisotropy (DA)	1.52 ± 0.10	1.37 ± 0.16	NS

### 3.3 SFX-01® treatment improves cortical bone mass and predicted indices of tibia strength

**Fig. 3:**
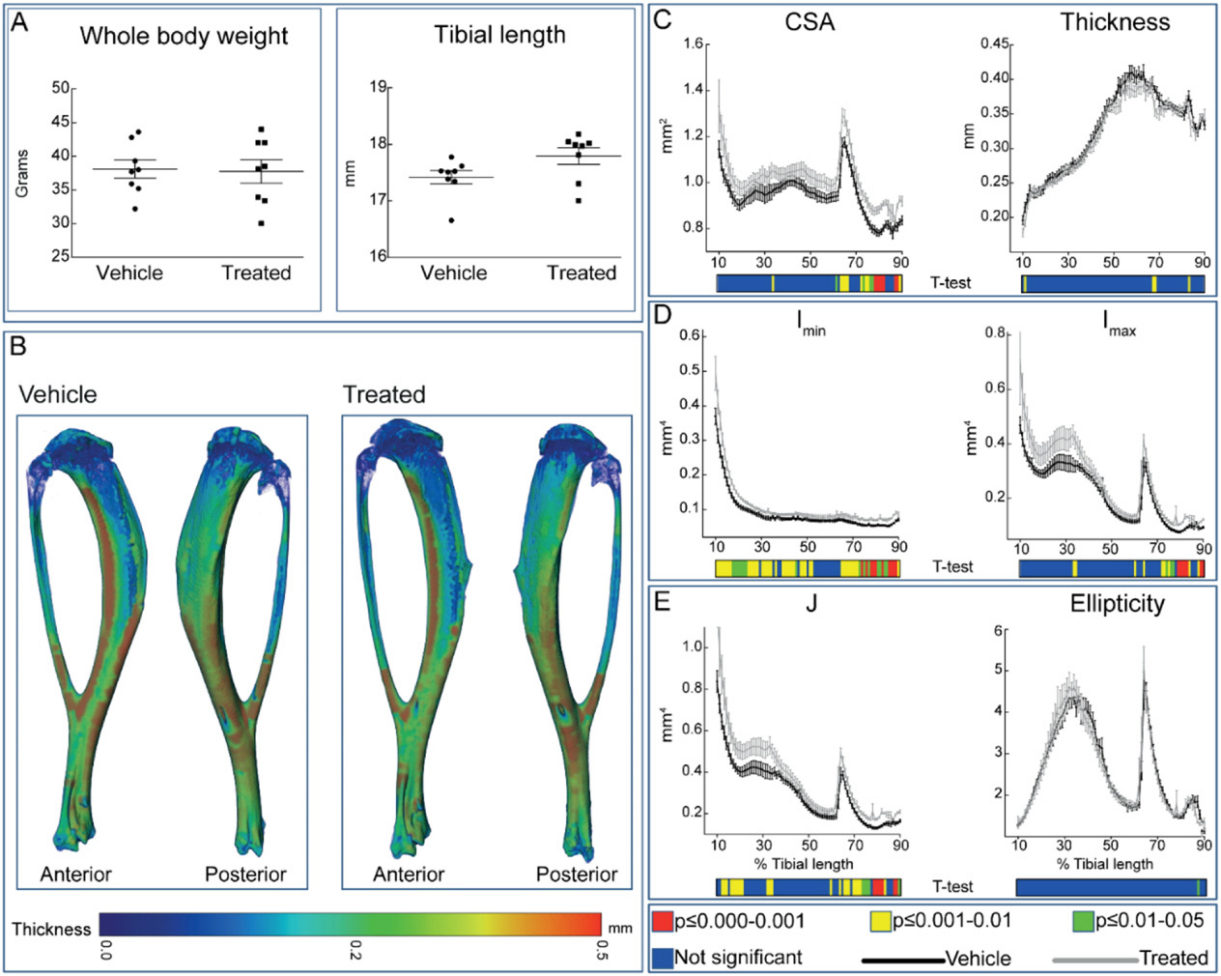
Cortical bone phenotype of vehicle (black) and SFX-01® (grey) treated mice. (A) Whole body weight and tibial length of vehicle and SFX-01® treated mice after 11 weeks of treatment. (*B*) Representative 3D Micro-CT colour-coded images of tibial cortical bone thickness. (*C*) Bone cross sectional area (CSA) and mean cortical thickness, (*D*) minimum and maximum second moments of area (I_min_ and I_max_ respectively) and (*D*) ellipticity and J (resistance to torsion) of vehicle and SFX-01® treated STR/Ort mice. Whole bone analyses of cortical bone between 10 and 90% of total tibial length, excluding proximal and distal metaphyseal bone. A two-sample *t*-test was used to compare means between vehicle KO and SFX-01® treated STR/Ort mice. Line graphs represent means ± SEM. Group sizes were *n* = 8 for vehicle and treated mice. Graphical heat map summarises statistical differences at specific matched locations along the tibial length, representative of overall effect of SFX-01® treatment. Red *p* ≤ 0.000–0.001, yellow *p* ≤ 0.001–0.01, green *p* ≤ 0.01–0.05 and blue *p* ≥ 0.05.

To determine whether osteotrophic effects of SFX-01® in STR/Ort mice extended to the bone cortices we undertook whole-bone tibia cortical analysis. We found no significant effect of SFX-01® treatment on body weight or tibial length (Fig. 3A) in STR/Ort mice. Nonetheless, cortical cross sectional area (CSA) was greater at many locations along the tibia length in SFX-01®-treated STR/Ort mice and reached statistically significant levels in the distal-most tibial region, from ~ 60% of length (Fig. 3C). Mean cortical thickness was only altered in small regions at the proximal and distal ends (Fig. 2B–C).

To provide an estimate of how these changes in cortical bone area modify tibial resistance to bending forces, we also calculated the second moment of area around minor (I_min_) and major axes (I_max_) as well as predicted resistance to torsion (J) and tibial shape as measured by ellipticity. These data showed that both I_min_ and I_max_ in tibiae of SFX-01® treated STR/Ort mice deviate from patterns observed in vehicle treated mice; I_min_ was significantly greater along almost the entire tibial length (apart from a region ~ 50–60% of length) and I_min_ was significantly elevated toward the distal tibia of STR/Ort mice treated with SFX-01®. Tibial shape as indicated by ellipticity was unaffected, yet predicted resistance to torsion (J) was significantly elevated in the SFX-01® treated mice at many locations mainly in proximal and distal tibia of STR/Ort mice. These data indicate that SFX-01® treatment in the STR/Ort model of spontaneous OA leads to significant enhancement in cortical bone area, I_min_ and I_min_ that consequently lead to enhanced predicted index of torsional, bending strength.

### 3.4 SFX-01® does not modify indices of natural OA in joints of STR/Ort mice

To establish the effect of SFX-01® treatment on natural OA development, as assessed by scoring of AC lesion severity, we investigated different components of tibiofemoral complex in STR/Ort mice. OA develops predominantly in medial tibia plateau of STR/Ort mice [10]. We therefore scored both OA mean and maximum grades in both medial and lateral joint compartments and found that neither mean nor maximum OA scores were modified by SFX-01® treatment in STR/Ort mice.

**Fig. 4:**
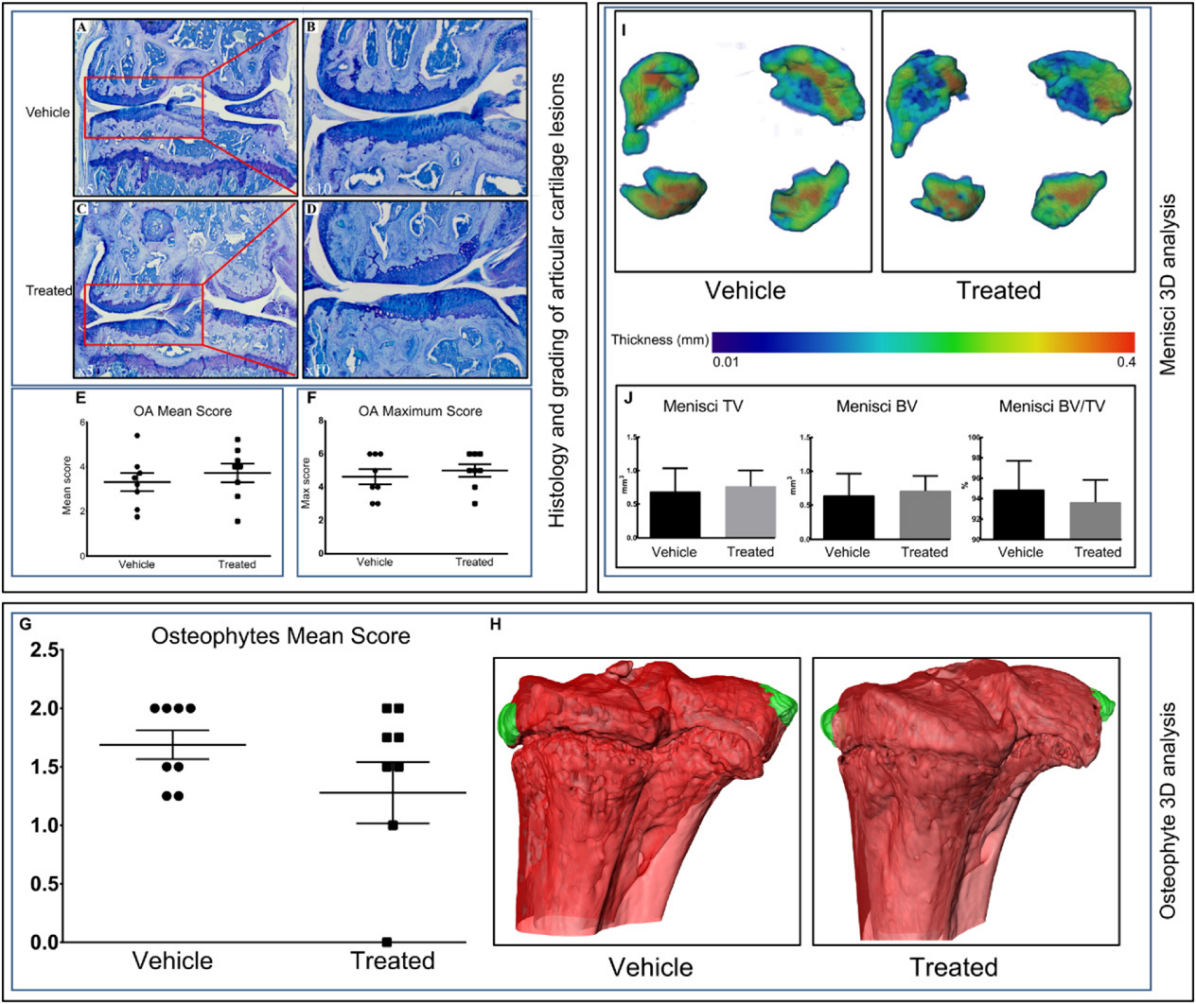
SFX-01® treatment does not modify different compartments of tibiofemoral complex. (*A–C*) (lower-power) and (*B–D)* (higher-power), toluidine blue stained sections of joints from vehicle (*A–B*) and SFX-01® (*C–D*) treated STR/Ort mice showing locations of naturally occurring lesions in the articular cartilage of the medial femur compartment of the tibiofemoral joint. Both vehicle and treated groups exhibited localized lesions. (*E)* and (*F)*, mean and maximum ± SEM lesion severity scores in each compartment of vehicle joints (circle) and treated joints (square). Osteophytes develop in both SFX-01® and vehicle treated STR/Ort mice; toluidine blue stained sections were scored to provide (*G*) mean number of osteophytes. (*H*) MicroCT was used to produce representative 3D images of osteophytes (green) in vehicle and SFX-01® treated mice. (*I*) Representative 3D Micro-CT colour-coded images of meniscal phenotype and (*J*) *ex vivo* analyses to determine TV, BV and BV/TV. Group sizes were *n* = 8 for vehicle and treated mice.

We also examined the presence of osteophytes using toluidine blue stained sections scored according to a grading system described previously [32] (Fig. 4G–H). Our data show that osteophytes developed similarly in each of the compartments of the knee joint in both SFX-01® and vehicle treated STR/Ort mice (Fig. 4H) and that neither the presence or severity of osteophytes was modified by SFX-01® treatment (Fig. 4G). Similarly, we found by microCT that SFX-01® treatment did not alter the overall thickness (Fig. 4I) nor the TV, BV and BV/TV of the menisci in STR/Ort mouse joints (Fig. 4J). Taken together, these data show that SFX-01® treatment did not modify these indices of OA severity, namely AC lesions, osteophytes or mineralised meniscal mass and architecture in STR/Ort mice.

### 3.5 SFX-01® treatment affects bone remodelling *in vivo* and *in vitro*

**Fig. 5:**
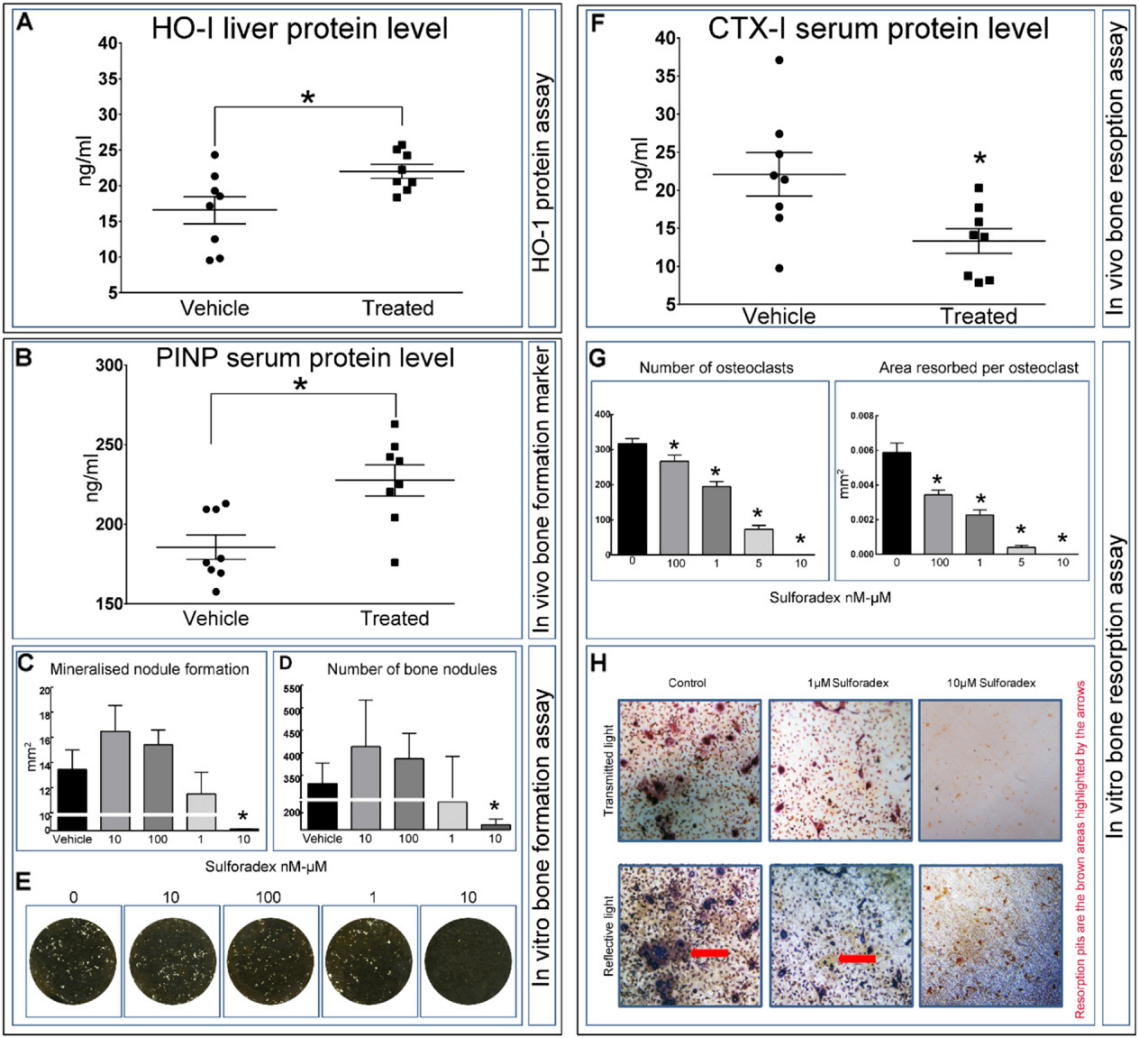
SFX-01® treatment activates downstream target (HO-1: heme oxygenase-1) and controls bone remodelling *in vivo* and *in vitro*. (*A*) HO-1 protein level in liver measured by ELISA. (*B) In vivo* blood serum protein level of procollagen type 1 N-terminal propeptide (P1NP: bone formation marker). (*C–D)* SFX-01® inhibited bone nodule formation at the highest dose only; no effects were seen at lower concentrations. (E) Representative whole well scans of osteoblast cell layers treated with different concentration of SFX-01®. (*F) In vivo* blood serum protein level of serum collagen type 1 cross-linked C-telopeptide (CTX-1: bone resorption marker). (*G)* SFX-01® dose-dependently inhibits osteoclast formation and resorptive activity compared to vehicle. (H) Representative transmitted and reflective light images of osteoclasts treated with 1–10 μM SFX-01®. Graphs represent means ± SEM. Statistical comparisons: * denotes *p* ≤ 0.05.

To explore the mechanism underpinning the SFX-01®-induced modifications in bone mass and architecture in STR/Ort mice, we next examined both hepatic markers of classic downstream antioxidant, anti-inflammatory targets of native SFN and serum markers of bone remodelling *in vivo.* To assess whether SFX-01® targets the classic downstream antioxidant, anti-inflammatory targets of native SFN, we first measured levels of hemeoxygenase-1 (HO-1; a downstream target of the nuclear factor, erythroid-derived 2-like 2 (Nrf2) promoter [34]. Our data showed that hepatic HO-1 protein levels were significantly upregulated by SFX-01® treatment (Fig. 5A; *p* ≤ 0.05), suggesting its *in vivo* targeting of mechanisms resembling SFN.

To assess the effect of SFX-01® on new bone formation *in vivo*, we measured serum levels of procollagen type I NH2-terminal propeptide (PINP). As shown in Fig. 5B, PINP levels were significantly elevated in response to SFX-01® treatment in STR/Ort mice (*p* ≤ 0.05). In cultured primary osteoblasts, 10–100 nM SFX-01® produced modest changes in both area and number of mineralised nodules, however, neither reached levels of statistical significance (Fig. 5C and D). Treatment with 10 μM SFX-01® almost completely abolished mineralised bone nodule formation (Fig. 4C–E; *p* ≤ 0.05).

We also found that serum levels of C-telopeptide of type I collagen (CTX-I), a bone resorption marker was significantly decreased in SFX-01® treated STR/Ort mice compared with vehicle-treated controls (Fig. 5F; *p* ≤ 0.05). In primary osteoclast cultures SFX-01® treatment (100 nM, 1 μM, 5 μM, 10 μM) dose-dependently reduced osteoclast number and resorption by up to 100% (Fig. 5G–H; *p* ≤ 0.05). Together, these data show that SFX-01® likely activates classic downstream target protein to exert its actions in STR/Ort mice and that these responses evoke enhanced rates of remodelling with enhanced bone formation *in vivo* and decreased indices of bone resorption both *in vivo* and *in vitro*.

## 4 Discussion

There is a lack of effective, disease-modifying pharmacological drugs available to treat OA and existing treatments that target symptoms are often inadequate. Non-pharmacological treatments, including physiotherapy and exercise, are ineffective for OA management and joint replacement remains often the only treatment offered to patients reaching end-stage OA [35], [36]. Previous studies have used different food-derived antioxidant pharmacological agents for the prevention or reversal of chronic degenerative disorders including OA. Among these agents, SFN has shown promising protection through cell cycle arrest, apoptotic, anti-angiogenic and anti-inflammatory actions [19], [37]. SFN is, however, unstable.

Herein, we explored whether a stabilised form of SFN, namely SFX-01® modifies gait and bone architecture, and slows/reverses the destruction of AC during spontaneous progression of OA in STR/Ort mice. STR/Ort mice have been used previously in interventional studies aimed at identifying targets that prevent and protect against AC lesions in OA joints. These have shown the importance of MMPs in AC degradation in STR/ort mice by administration of Ro 32-3555, an orally active collagenase selective inhibitor, which protected against OA development [38]. In addition, intra-articular injections of CRB0017 (anti-ADAMTS5 antibody) dose-dependently slowed OA progression in STR/Ort mice across a slightly older 5–8 month age range than we used [39]. Our data reveal that treadmill task non-compliance and the development of asymmetric gait (most obviously demonstrated in uneven distribution in hind limb paw area in left/right limbs), which is normally linked to OA [14] in vehicle-treated STR/Ort mice, failed to emerge in SFX-01®-treated mice; fore-limb asymmetry followed similar patterns. Our previous studies comparing gait in STR/Ort and sex-/age-matched non-osteoarthritic CBA mice (closest commercially available parental strain) showed that unlike STR/Ort, CBA mice show full treadmill task compliance and that paw area and gait asymmetries were the best predictor of age-related OA onset and severity.

In contrast, we found that histologically-graded OA in articular cartilage was unmodified by SFX-01® treatment and hence unlikely linked to any correction of OA-related gait asymmetry. The OA scoring system we use is closely aligned to an internationally-recognised system devised by Osteoarthritis Research Society International (OARSI) for grading of OA joints in animal models. It was devised to be more sensitive across milder OA grades and applicable with more consistently by less experienced observers than the original system proposed by Mankin [40], [41], [42], [43]. Application of this scoring system to different OA murine models was highly reproducible [13] and we have used it to grade OA in mice of this age group previously under both similar and other conditions [14], [44], [45]. Its application to early OA stages in have also been found to correlate with mRNA levels of molecular markers of OA development in STR/Ort mouse cartilage [46]. Thus, our studies which failed to identify any beneficial effect of SFX-01® in articular cartilage make it highly unlikely that these molecular signatures will have been affected by the very long-term SFX-01® treatment.

We have previously reported that gait changes in STR/Ort mice are not necessarily accompanied by any measurable local inflammatory joint pain, such as that induced by intraarticular injection of Freund's complete adjuvant (CFA). This indicates that hind limb paw area measurement may be a useful, non-invasive monitoring tool that likely reflects structural, rather than local inflammatory pain-related, changes in the joint. Previous studies have reported the presence of low thresholds for mechanical stimuli, pain and hyperplasia in patients with OA suggesting a non-nociceptive pain element. These observations have provided evidence to support the notion that neuropathic pain mechanisms contribute to the pain experience of the OA population [47], [48], [49]. Interestingly, recent studies have suggested that SFN is an effective treatment for other forms of neuropathic pain [50]. Our data indicate that SFX-01® treatment corrects the OA-associated asymmetries in gait that occur in untreated STR/Ort mice, without modifying articular joint scores, suggesting that SFX-01® targets a component of OA-linked neuropathic pain that is hitherto unidentified in the spontaneous OA that occurs in these mice.

Our detailed analyses of different components of tibiofemoral complex as well as entire tibial cortical bone reveal that SFX-01® treatment of STR/Ort mice produces an enhancement in trabecular bone mass in the metaphysis, an expansion of the volume but not significant alteration in bone mass in the epiphysis without any modifications in the thickness of the subchondral plate. We consider these differences to be dominated by the more marked volumetric increases in the epiphysis *vs.* the metaphysis. Previous studies have shown that subchondral bone particularly is important in the pathogenesis of OA [3], [4], [5], [6], [51] and that this should be targeted by OA-modifying drugs [52]. Several studies have reported, using different bone modifying agents in animal models and in cells *in vitro* including oestrogen [53], bisphosphonate [54], intermittent parathyroid hormone [55] and strontium ranelate [56] are capable of modifying subchondral bone remodelling and OA progression. It is also interesting to note that some clinical trials using bone antiresorptive and anabolic drugs have suggested that OA-linked pain is improved in response to these selective bone modifying agents [57]. Our findings strengthen the possibility that these bone-targeting therapies may have some merit, since we find that SFX-01® treatment both modifies bone architecture in the STR/Ort mice and likely reduces OA pain and improves gait, without seemingly influencing the AC lesions severity in the joints of these mice. It is important, nonetheless, that long-term pain relief without directly targeting AC lesion development, may itself predispose joint to alterations in their mechanical loading environment and thus hasten AC deterioration [58]. Osteophyte formation is also an important characteristic of OA in humans and in mice [59]. We, however, found no effect of SFX-01® treatment on osteophytes and meniscal mineralised tissue mass and architecture.

Our data also show that treatment with SFX-01® leads to a significant enhancement in cortical bone mass in STR/Ort mice that improves predicted indices of torsional, bending strength. These significant region-specific effects of SFX-01® on cortical bone mass and architecture are possibly due to differences in region specific transcription factor expression as described previously [60]. We also assume, due to structural differences, that load-induced strain distribution and bone remodelling be different at different anatomical locations. The likelihood that these effects are achieved by direct actions on bone cells is supported by our data showing both anabolic and anti-catabolic actions of SFX-01® treatment *in vivo*, evidenced by alteration in serum markers, as well as changes in isolated primary osteoblast and osteoclast-like cell behaviour in response to exogenous SFX-01® *in vitro*. Indeed, our results with SFX-01® are in close agreement with previous studies in which SFN has been reported to elicit both anabolic and anti-resorptive properties [22]. Although our *in vitro* osteoblast assays show a beneficial trend of SFX-01 treatment, they did not reach statistical significance and hence do not match perfectly with elevated serum PNIP levels. In our study, STR/Ort mice were treated with daily SFX-01® for 3 months whereas primary osteoblasts were only exposed to SFX-01® for days and thus longer exposure to SFX-01® *in vivo* might underlie these differences. It is also possible that SFX-01® affects bone formation indirectly and additional studies are needed.

It is important to emphasize that the animals used in this study were 26 weeks of age and according to a wealth of historical data, already osteoarthritic. It is therefore possible that earlier treatment of STR/Ort mice with SFX-01® prior to OA initiation (pre-18 weeks of age) might exert additional protective effects on OA-related cartilage degradation and other joint markers of OA severity. This is highly likely since previous studies have reported that SFN can inhibit the activation of a variety of effector and initiator caspases, critically involved in both intrinsic and extrinsic pathways of chondrocyte apoptosis and cartilage damage in arthritic diseases [61].

In conclusion, we report that SFX-01® improves bone microarchitecture *in vivo,* produces corresponding changes in bone cell behaviour *in vitro* to enhance indices of bone mechanical strength and produces greater symmetry in gait, without marked effects on cartilage lesion severity in STR/Ort osteoarthritic mice. Our findings support both osteotrophic roles and novel beneficial gait effects for SFX-01® that appear to be independent of articular cartilage lesion development in this model of spontaneous OA.

## Competing interests

The authors have no conflict of interest to declare.
